# A GIS Approach to Modeling the Ecological Niche of an Ecotype of *Bouteloua curtipendula* (Michx.) Torr. in Mexican Grasslands

**DOI:** 10.3390/plants14142090

**Published:** 2025-07-08

**Authors:** Alma Delia Baez-Gonzalez, Jose Miguel Prieto-Rivero, Alan Alvarez-Holguin, Alicia Melgoza-Castillo, Mario Humberto Royo-Marquez, Jesus Manuel Ochoa-Rivero

**Affiliations:** 1 Campo Experimental Pabellon, Instituto Nacional de Investigaciones Forestales, Agrícolas y Pecuarias (INIFAP), Km 32.5 carr. Zac-Ags., Aguascalientes 20660, Aguascalientes, Mexico; 2 Facultad de Zootecnia y Ecología, Universidad Autónoma de Chihuahua, Periférico Francisco R. Almada Km. 1., Chihuahua 31125, Chihuahua, Mexico; 3 Campo Experimental La Campana, Instituto Nacional de Investigaciones Forestales, Agrícolas y Pecuarias (INIFAP), Km 33.5 carr. Chih-Ojinaga, Cd. Aldama 32910, Chihuahua, Mexico

**Keywords:** biogeography, ecological niche modeling, Chihuahuan desert, sideoats grama, *Bouteloua curtipendula*, grassland rehabilitation

## Abstract

The reliance on imported seeds for grassland rehabilitation in Mexico has led to increased costs and other difficulties in implementing grassland rehabilitation programs. Varieties need to be generated from local ecotypes that are outstanding in forage production and their response to rehabilitation programs. However, the scarcity of occurrence records is often a deterrent to niche and distribution modeling, hence the need for an approach that overcomes such limitations. The objectives of this study were (1) to develop a geographic information system (GIS)-based approach to determining the population distribution of a promising ecotype of *Bouteloua curtipendula* (Michx.) Torr. for grassland rehabilitation in the Chihuahuan Desert, Mexico; (2) to identify the edaphoclimatic variables that define the ecotype’s distribution; and (3) to develop models to determine the potential area for the use of the ecotype in grassland rehabilitation. The challenge for the present study was that only one georeferenced collection site of the ecotype in Chihuahua was available for use in the construction and calibration of the models. GIS software 10.3 was used to develop two potential distribution models: Model A, with variables obtained directly from a vector climate dataset, and Model B, with derived variables. A field work methodology was developed for the validation process using a georeferenced digital mesh and the nested sampling method modified by Whittaker. The information was analyzed with 10 non-parametric statistical tests. The two models had an overall accuracy and sensitivity level greater than 70% and a positive predictive power greater than 80%. The predicted population distribution areas in Chihuahua (18,158 ha) in the form of discontinuous patches cohered with those in previous reports on the distribution form of *B. curtipendula*. The edaphoclimatic variables influencing ecotype distribution were soil type, average minimum and maximum temperature in January, average maximum temperature in June, average minimum temperature in July, and average precipitation in August. The sensitivity analysis showed soil type as an important variable in defining the ecotype’s distribution. Considering soil as the main predictor variable, the potential rehabilitation area where the ecotype may be used was estimated at 7,181,735 ha in the Chihuahuan Desert region. The study developed and validated an approach to modeling the ecological niche of an ecotype of commercial interest, despite severe limitations in the number of georeferenced sites available for modeling. Further study is needed to explore its applicability to grassland rehabilitation in the Chihuahuan Desert and the study of rare and understudied ecotypes or species in other settings.

## 1. Introduction

Grassland areas are among the world’s major ecosystems, covering almost a third of the Earth’s land surface [1] and serving as the main type of land for grazing and habitat for numerous species [2]. Grasslands are essential to human subsistence and biodiversity conservation because of their key role in livestock production, as well as in hydrological cycling, carbon sequestration, nutrient cycling, erosion control, pollination, and climate mitigation [1,3,4]. Unfortunately, grasslands are among the most critically endangered ecosystems globally, requiring restoration because of the decline over the past century due to environmental and anthropogenic disturbances [1,4,5]). In Mexico, experts have identified the need for partial reforestation of 5.7 million ha and complete reforestation of 32.4 million ha [6,7] to reverse desertification caused by high-risk agriculture and overgrazing [7].

The reliance on imported seeds for grassland rehabilitation in Mexico has led to increased costs and other difficulties in implementing grassland rehabilitation programs. The use of ecotypes not adapted to the local conditions has reduced the success of these programs, thus it is imperative that varieties be generated from local ecotypes that are outstanding in terms of forage production and their response to rehabilitation programs [8].

*Bouteloua curtipendula* (Michx.) Torr. or sideoats grama is a perennial species that has been successfully used in livestock production. Present in diverse environments, with a distribution from Canada to South America, it is found in 24 Mexican federal entities [9,10,11]. *B. curtipendula* is a tillered-type species with deep roots and fibrous growth that adapts to a wide diversity of shallow stony soils 15 to 50 cm deep, with slopes of 1 to 60%. As such, it contributes greatly to infiltration and sediment reduction in moderately saline to slightly acidic soils [10,12]. Present in areas with precipitation of 200 to 700 mm, *B. curtipendula* is tolerant to drought and moderate shading and, in the dormant stage, resistant to fire and spring floods [10,13].

When a species is introduced into a new environment, local agro-climatic conditions interacting with the genetic variations of the introduced population contribute to creating new ecotypes, which are defined as populations that are genetically adapted to specific environmental conditions [14]. *Bouteloua curtipendula* ecotype B-31 is one of the ecotypes that have been collected around the city of Parral, Chihuahua (26°55′14.38″ latitude north and 105°48′25.35″ longitude) [9,15]. This area has a hilly topography and a temperate semi-dry climate with warm and rainy summers (BS 1 kw (w)), annual precipitation of 400 to 500 mm, and winter precipitation of 10.8 to 13.5 mm. The average annual temperature ranges from 15 to 18 °C [16]. Based on ex situ evaluations of different *B. curtipendula* populations, the B-31 ecotype can reach a production of 1.7 Mg DM ha^−1^, with an establishment of 22 plants m^−2^ in the second year after sowing [17], making it a genetic resource with a high potential for rehabilitating grassland areas. However, its potential population distribution and the environmental conditions to which it adapts are unknown. Vallejo-Trujillo et al. [14] stress the need for environmental modeling for defining ecotypes that considers the complex interaction of a large number of environmental predictors. In this regard, the scarcity of occurrence records has often been considered a deterrent to niche and distribution modeling, hence the need for an approach that overcomes such a limitation.

Simulation models may be used to define outstanding ecotypes and their population distribution; they aid in delimiting collection areas as well as areas where the soil–climate–genotype conditions are appropriate for the species [18,19]. Ecological niche modeling (ENM), also referred to as habitat suitability modeling (HSM) and species distribution modeling (SDM) [20,21,22,23], despite their distinct differences [24], aims to identify places suitable for the survival of a species by identifying the environmental requirements of the species [24]. ENM estimates the actual or potential areas of distribution or favorable habitats for a species based on its observed presences and absences. Presence-only and presence–absence studies, being rather different [24], require algorithms [21] that are conceptually appropriate to them [25].

Most often, large datasets from the collections available in regional, national, and/or international databanks are needed in ENM studies that use such tools as Max-Ent [26]. In those studies, the data are partitioned using a one-time data-splitting approach [27], with one portion used to construct and calibrate a model and the other to validate it [28,29,30]. The use of a small sample size is believed to increase model variance and bias [31]. In their evaluation of predictions at three sample sizes (100, 30, and 10 records), Wisz et al. [32] reported that model accuracy decreased and variability increased with decreasing sample size, and with small sample size (*n* < 30), no algorithm predicted consistently well. They therefore recommend that predictions based on small sample sizes be treated conservatively and their use restricted to exploratory modeling.

However, in the case of rare or endangered species, the use of a small number of occurrence samples, though not considered ideal, has been helpful in determining species distribution [18,33,34]. A study evaluating sampling size has shown that the modeling ability is largely influenced by species ecological characteristics, independent of sample size, and that species with compact spatial distributions tend to be easier to model than species that are widespread in geographic and environmental space [33].

Several techniques for modeling species with few occurrences have emerged [35]. Among them is the use of ensembles of small models (ESMs), i.e., several simple models with just a few variables each to create an ensemble prediction [36,37]. It has been recommended that when the sampling size is small, the number of predictors in a model be limited to ~10 presences per coefficient [31]. Another technique is the use of multi-species distribution models (MSDMs), in which multiple species are modeled at once and statistical “borrowing of strength” is employed so that information from more numerous species helps estimate coefficients for less numerous ones [31,38,39]. MSDMs have been used to model species with zero or very few occurrences [40]. However, some authors recognize that the problem of modeling extremely rare species is not easily remedied by these approaches and sometimes simpler methods are best [31,41]. They believe that researchers should consider what they already know about their species of interest and identify the goal of their modeling effort before deciding on their modeling approach [31].

In the case of the present work, only one georeferenced collection site of *B. curtipendula*. B-31 in Chihuahua [8] was available for use in modeling. Therefore, the challenge was to develop a methodological approach that would allow ENM at the ecotype level, considering each of the modeling stages (i.e., construction, calibration, validation, and sensitive analysis), in order to determine the actual and potential population distribution of the ecotype and the edaphoclimatic characteristics determining its establishment.

The objectives of this study were (1) to develop a GIS-based approach to determining the population distribution of *B. curtipendula* (Michx.) Torr B-31, a promising ecotype for grassland rehabilitation in the Chihuahuan Desert, Mexico; (2) to identify the edaphoclimatic variables that define its distribution; and (3) to develop models to determine the potential area for rangeland rehabilitation using this ecotype. This work was part of the Project on Varietal Registry of New Elite Ecotypes and Generation of Technology for Pasture Seed Production of the National Institute of Forestry, Agricultural and Livestock Research (INIFAP) of Mexico [8]. One of the research activities of this project was the development of population distribution models of elite *B. curtipendula* ecotypes in Chihuahua, Mexico.

## 2. Results and Discussion

### 2.1. Predicted Population Distribution

**Figure 1:**
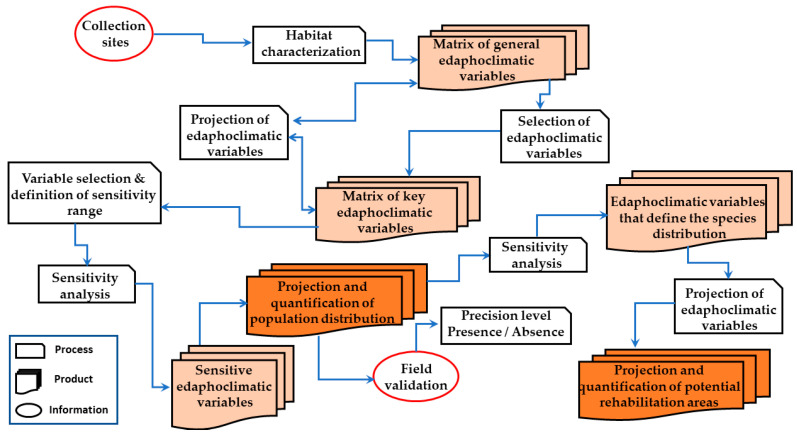
Methodology applied in the model construction, calibration, and validation of the population distribution model of the B-31 ecotype of *Bouteloua curtipendula* (Michx.) Torr. in the grasslands of Chihuahua State, Mexico.

As a result of the processes shown in Figure 1, two types of predicted population distribution models were generated: (1) a model with direct variables (Model A) and (2) a model with derived variables (Model B) (Table 1). For both models, Foezem soil best described the ecotype habitat. In Model A, the climatic variables that best described the ecotype habitat were the average precipitation in the month of August, average maximum temperatures in January and June, and average minimum temperatures in January and July, while in Model B, they were the average precipitation in the month of August, average daytime temperatures in January and June, and average nighttime temperature in January. On the other hand, altitude was not relevant in the description of the ecotype. This differs from the findings of Alvarez-Holguin et al. [26], which indicated that elevation was the variable with the highest contribution to the environmental niche model for the *B. curtipendula* species. Our results provide additional evidence that habitat description at the ecotype level can differ from that at the species level.

**Figure 2:**
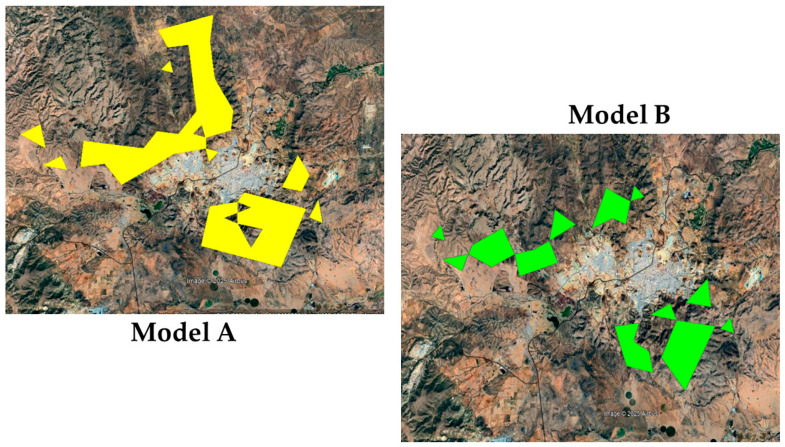
Predicted distribution population of *Bouteloua curtipendula* (Michx.) Torr. ecotype B-31 in the grasslands of Chihuahua State, Mexico. The predicted population distribution considering direct variables (Model A) is in solid yellow and the predicted population distribution considering derived variables (Model B) is in solid green.

The distribution pattern generated by the two models showed a predicted population distribution in the form of discontinuous patches (Figure 2). In Model A, the patches were of greater amplitude than those in Model B. However, both predicted a population distribution in the same areas and only differed in the magnitude of territorial extent (Figure 2).

### 2.2. Validation of Predicted Population

The quality analysis of the field data gathered during the validation phase showed that one of the quadrants located outside of the projected surface (i.e., predicted absence sites) was 250 m from the potential distribution of Model B. The eight field sub-sites in this quadrant were thus not considered in the statistical analysis. In the case of the field sites located within the projected surface for each model (i.e., predicted presence sites), the quadrants were exactly within the areas predicted by each model; therefore, the sub-sites of presence in said areas were classified as “sensu strictu”. The quadrants in the areas of overlap between models were geographically located correctly in these areas; only the sub-sites reported as presence were considered as “sensu lato” data (data at the point of interaction between areas predicted by both models), and these were added to the sensu strictu data. The confusion matrix thus yielded a total of 46 field data points for Model A and 48 data points for Model B (Table 2).

The results of the statistical tests showed that in terms of overall accuracy, degree of error, and sensitivity, the two models were similar (Table 3). However, as can be seen in the specificity test (i.e., the probability that a case other than X will be correctly classified as false), Model A showed higher probability than Model B to correctly classify a false X (0.87 vs. 0.67). The positive and negative Likelihood Rates (LRs) of Model A were 5.56 and 0.30, respectively; for Model B, they were 2.27 and 0.36, respectively (Table 3).

Another statistical test that showed a numerical difference between the two models was the odds ratio test. This test expresses the relationship between correctly assigned cases and incorrectly assigned cases. Model A obtained a value of 19, while Model B had a value of 6. In general, both models presented an accuracy greater than 70% (Table 3). The error of less than 30% in the models indicates that for every 10 sites defined as sites of ecotype presence, 8 sites will be correct in Model A and 7 sites in Model B.

As mentioned previously, the two models presented a predicted population distribution pattern in the form of patches, and the difference between them was the shape and extent of these patches (Figure 2). The results are congruent with the findings of Laughlin [42], who reported that in a study of *B. curtipendula* in Pennsylvania, USA, the population distribution pattern was limited to small and isolated areas of less than 0.3 ha and separated from each other by distances greater than 16 km. As explained by Laughlin [42], the diaspores of *B. curtipendula* can be dispersed only a few meters by wind and the seeds are destroyed in the rumens of grazing animals; hence, long-distance dispersal is mainly by adhesion to animal fur.

On the other hand, irregular vegetation patterns in semiarid grasslands have been related to autocorrelation between some soil physical parameters [43]. Some authors [44,45,46,47,48] mention that organic matter, apparent density, and availability of phosphorus in soil, parameters directly linked to the presence/absence of vegetation, do not show a uniform distribution in scrubland areas, which is why scrubland landscapes are more heterogeneous in nature than grasslands. Low organic matter and high apparent densities characterize intershrub areas, resulting in a low infiltration capacity and increased susceptibility to the erosive power of surface flow. Laughlin [42] concludes that due to the absence of long-distance dispersers and the dispersed distribution of suitable edaphic conditions, *B. curtipendula* is “trapped” in small sites that are shrinking due to invasion by woody plants.

### 2.3. Environmental Factors That Shape the Distribution of the Ecotype

A sensitivity analysis was performed to compare the contributions of the environmental variables and assess the importance of each in determining the distribution of the ecotype [49,50,51,52,53]. This analysis was carried out considering the variables making up Model A, since this model showed the highest values in overall accuracy, specificity, and positive predictive power and the lowest negative LR.

**Figure 3:**
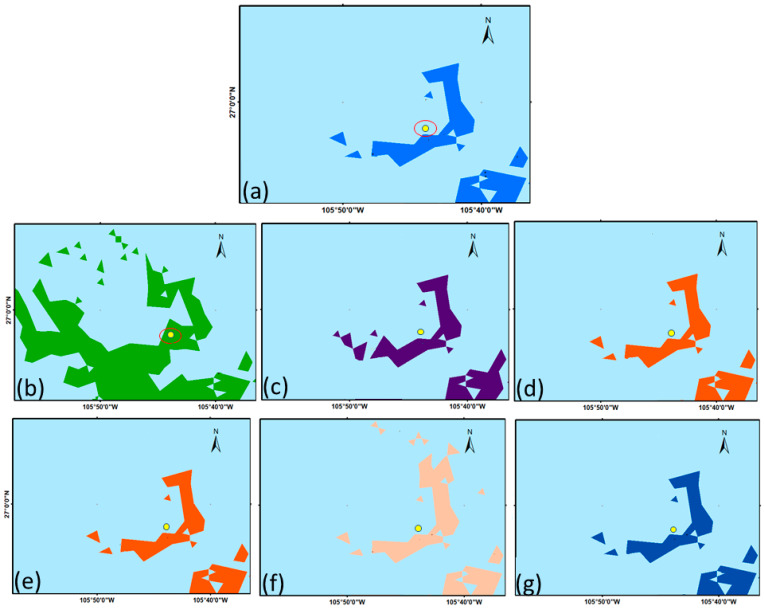
Predicted distribution population of *Bouteloua curtipendula* (Michx.) Torr. ecotype B-31 in the grasslands of Chihuahua State, Mexico, considering all the variables of Model A (**a**), Model A without soil type (**b**), Model A without maximum temperature in June (**c**), Model A without maximum temperature in January (**d**), Model A without minimum temperature in January (**e**), Model A without minimum temperature in July (**f**), and Model A without August precipitation (**g**). The yellow dot shows the location of one of the quadrants used to validate the predicted absence sites.

First, we used GIS to estimate the total area of the population distribution in Model A, considering all the variables that best described the habitat of the ecotype in the state of Chihuahua. Then, each of the six variables was eliminated one at a time from Model A. Hence, six submodels were generated (Figure 3). The predicted surface area was calculated for each submodel to estimate the impact of excluding a variable from the base model (Table 4). This provided a way to assess how changes in the model inputs (variables considered) affected the model’s output (predicted distribution area).

The quadrants sampled during the field validation phase of Model A were projected onto each submodel (Figure 3), which made it possible to use the predicted presence/absence information (Table 2) to perform two statistical tests on each submodel: negative LR test and True Skill Statistic (TSS). The TSS, as applied in presence–absence predictions, provides a threshold-dependent measure of accuracy [54]. As can be seen in Table 4, submodel 1, which excluded soil type, obtained a negative LR value of 0.42 and a TSS of 0.48 (vis-a-vis Model A values of 0.30 for negative LR and 0.61 for TSS). The increase in the negative LR value and decrease in accuracy value in submodel 1 were due to the fact that the exclusion of soil type resulted in a 200% increase in the distribution area, causing one of the quadrants sampled in the field that validated predicted absence (Figure 3b) to fall within the predicted distribution area (i.e., predicted presence). In the case of the climatic variables, their exclusion in submodels 2 to 6 showed values of 0.30 and 0.61 for negative LR and TSS, respectively (Table 4). These values were equal to those obtained by Model A. As seen in Figure 3 and Table 4, the surface areas predicted by the submodels that excluded the climatic variables were very similar to that predicted by Model A. These results suggest that soil type, rather than climate variables, was most influential in driving the model’s behavior.

The only climatic variable whose exclusion resulted in an increase of 30% in the area of distribution was the average minimum temperature in July (Table 4, submodel 5). However, this increase in surface area did not cause a change in its accuracy and negative LR values when compared to Model A. Previous studies have shown that temperatures, such as night temperatures, can inhibit physiological processes in plants [55,56]. Temperatures can have an asymmetric effect on the growth of grasslands [57,58,59,60] due to the increasing photosynthetic enzyme activity related to daytime warming, while the rising minimum temperature (T min) could hinder grassland growth due to the negative effect of enhanced autotrophic respiration [57]. Wang et al. [61] reported on the asymmetric impacts of summer T min and maximum temperature (T max) on the net primary productivity (NPP) of vegetation, while An et al. [62] mentioned that there is a higher probability of nighttime extreme high temperatures causing abnormal increases in NPP compared to daytime extreme high-temperature events.

**Figure 4:**
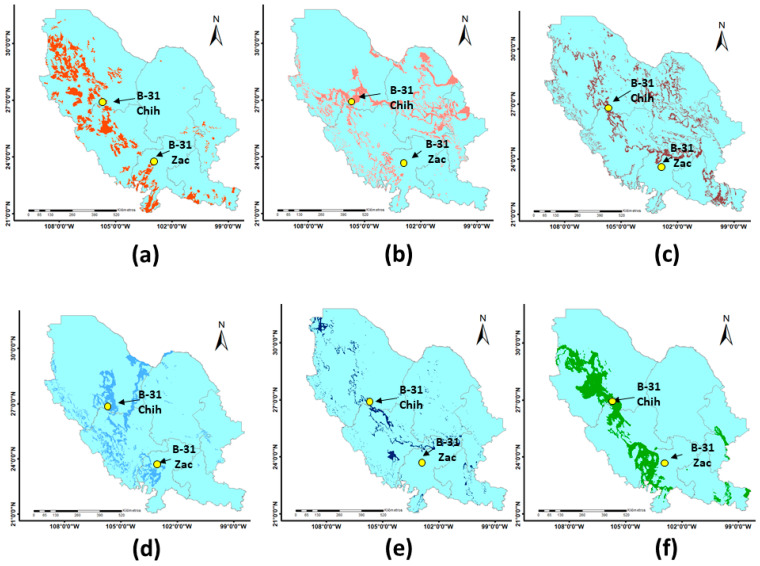
Projection of potential rehabilitation areas with *Bouteloua curtipendula* (Michx.) Torr. ecotype B-31 in the Chihuahuan Desert region considering each of the following variables as an independent predictor: (**a**) soil type, (**b**) maximum temperature in January, (**c**) maximum temperature in June, (**d**) minimum temperature in January, (**e**) minimum temperature in July, and (**f**) August precipitation. Yellow dots indicate the field locations of collection sites of ecotype B-31 in the states of Chihuahua and Zacatecas, Mexico.

To further observe the performance of soil and the other variables of Model A as independent predictors, they were projected individually onto the Chihuahuan Desert area (Figure 4) to see if their predicted population distribution would include a B-31 collection site located in that region in the state of Zacatecas. This collection site had not been used in any of the earlier modeling processes of the present study because it was outside the study area (i.e., Chihuahua State). It can be observed in Figure 4a that the Zacatecas collection site was within the distribution area projected with soil type as predictor variable. This adds certainty to the projections generated by the present study and supports our finding that soil is an important factor to consider when modeling the distribution of the B-31 ecotype. The maximum temperature in June was the climatic variable with the shortest distance between the predicted distribution area and the collection site (i.e., 849 m).

### 2.4. Potential of Bouteloua curtipendula (Michx.) Torr. Ecotype B-31 as a Genetic Resource for the Rehabilitation of Grasslands in the Chihuahuan Desert

The widespread use of native grasses is considered advantageous, as they tend to have lower fertilizer requirements, high drought tolerance, and high yield stability [63]. In a previous ex situ study [17], the B-31 ecotype was seen as having great potential for use in grassland rehabilitation; hence, we estimated the number of potential hectares where this ecotype could be established.

**Figure 5:**
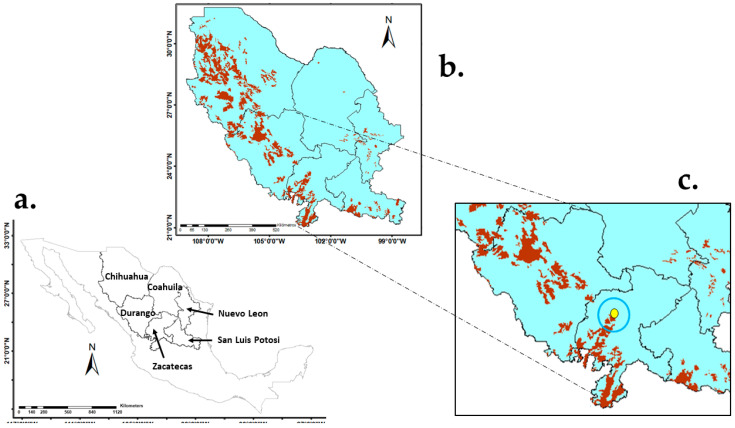
Projection of potential rehabilitation areas with *Bouteloua curtipendula* (Michx.) Torr. ecotype B-31 (brown areas) in the Chihuahuan Desert region (**a**,**b**). Field location (**c**) of a specimen of ecotype B-31 (yellow dot with blue circle) within the area predicted as having potential for rehabilitation in the Chihuahuan Desert, Mexico.

From the restoration point of view, it has been recommended [64,65,66] that the genetic material of a region remain in that area, even if it is widely distributed, in order to maintain stability in the ecosystems. For instance, Choi et al. [67] reported that several native populations with specific genetic structures were affected by the use of foreign seeds of the shrub *Lespedeza cuneata* (Dum. Cours.) G. Don during area restoration. We therefore ran the model considering the Foezem soil type, the main predictor variable according to the results of the present study (Table 4 and Figure 4), and quantified the potential use of B-31 for grassland rehabilitation only in areas in the region of the Chihuahuan Desert, which spans Chihuahua and five other Mexican states in northern Mexico (Figure 5a). The potential rehabilitation area was estimated to be 7,181,735 ha (Figure 5b). Unfortunately, because of the lack of sufficient georeferenced sites across the Chihuahuan Desert, it was not possible to perform any statistical analysis, and the study was able to only corroborate visually with the use of the collection site in Zacatecas (Figure 4).

It can be observed in Figure 5c that when the georeferenced collection site of this ecotype in the state of Zacatecas [17] was projected onto the predicted area in the Chihuahuan Desert region, its location was within the projected area for potential rehabilitation in the region. As stated earlier, this provides visual support for the adequacy of the projection and the relevance of soil as predictive variable. Further study with additional statistical analysis and field data from sites across the Chihuahuan Desert region is needed in this regard. However, the results of this study, along with related information from other studies, may be of immediate practical use for determining priority areas for collection efforts and pilot studies in grassland rehabilitation in the region.

### 2.5. Limitation of the Approach

Our study used presence-only data from one georeferenced collection site in model construction but considered both the presence and absence data gathered during the field validation. Presence-only data have been used in ENM [68] for envelope techniques, such as BIOCLIM [69], and similarity methods, such as Mahalanobis distance classification [70]. However, the use of sufficient data for both presence and absence to not only train but also test models has been recommended [71]. A sample size lower than 10 presences and 10 absences is believed to lessen predictive power [72]; hence, the recommendation is for test sets to include at least 15 occurrence and 15 absence points and for the minimum initial sample size of a dataset to be at least 50 occurrences and 50 absences. A common approach to cross-validation is splitting a single initial dataset into a training set of usually 70% of the data for model calibration and a test set of 30% of the data for assessment of model performance [73]. Collart and Guisan [71] recommend further study to determine the minimum dataset required for the optimal testing of models; they assert that while accurate predictions of species distribution are possible even with a low sample size, incorrect model evaluations may result from the use of small test sets.

Another limitation to consider is that our study used only scenopoetic variables (soil and climatic variables) and did not include an analysis of the role of biotic interactions in the ENM, although neighboring plants were identified. While studies have demonstrated the predictive value of the use of scenopoetic variables [74,75,76,77], the failure to consider the role of biotic interactions in ENM is considered an important limitation to reliable predictions of species’ distributions [78]. Interactions and dispersal disequilibrium can impede a species from fully occupying its potential niche. Hence, not including both scenopoetic and bionomic variables may lead to over-simplification. It is thus necessary to indicate qualifications and caveats in this regard [24].

In the present study, the challenge was to determine the potential distribution of an ecotype through the use of only one recorded presence site. The scarcity of occurrence records in cases like this is often attributed to factors such as species rarity, sampling difficulty, and the unavailability of electronic data [32]. Another contributing factor is the general belief that large datasets are needed for ENM. However, in order to ensure the conservation of rare and understudied species under climate change and other challenging conditions, it is sometimes necessary to conduct studies using whatever data are available, even if the data are not ideal. Such studies can contribute to fine-tuning collection and conservation efforts and thereby help remedy the problem of data paucity in ENM. This study contributes a GIS-based approach for studying an understudied ecotype that has potential use in rehabilitation efforts in the Chihuahuan Desert grasslands. Its applicability to other understudied ecotypes or species in other settings can be explored.

## 3. Materials and Methods

### 3.1. Study Area

**Figure 6:**
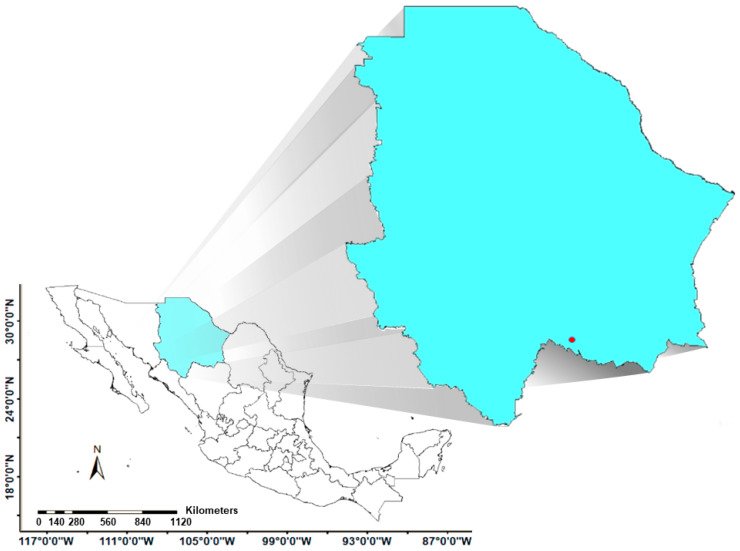
Study area and location of the collection site (red point) of *Bouteloua curtipendula* (Michx.) Torr. Ecotype B-31 in the state of Chihuahua, Mexico.

The study area covers the grasslands of Chihuahua State, Mexico (Figure 6). The natural grasslands that thrive in a wide range of ecological conditions in large areas of the physiographic provinces of the Sierra Madre Occidental and the sierras and plains of the north are among the most important grassland areas in the country. Grasslands, both natural and induced, cover approximately 59,390 km^2^, or 24% of the territory of Chihuahua, [16] at altitudes of 1200–2300 m. Most of them are at the foot of the mountain range, in semi-dry temperate climates with average annual temperatures of 12–18 °C and an average total annual rainfall of 300–600 mm. The rest are in the north and northeast of the state, with very dry or temperate desert climates having similar temperature ranges but with precipitation rates below 400 mm and some in places with very dry semi-warm climates with very low precipitation numbers and higher average annual temperatures reaching 19 °C. The most frequent soils on the sierra foothills, where grasslands thrive, are Regosol, Cambisol, and Feozem, with medium texture and limited by the bedrock to less than 50 cm of depth [16].

*B. curtipendula* (Michx.) Torr. B-31 was collected in an ecoregion dominated by xerophilous scrub and oak-pine forest in the southern part of Chihuahua (Figure 6) at 1834 amsl in a Foezem Luvico-type soil. During the field work, it was observed that the medium arborescent grassland site where ecotype B-31 was located had a high degree of disturbance due to the substantial coverage of shrub elements, mainly *Mimosa* L. spp., *Opuntia* Mill. spp., *Nolina texana* S. Watson., *Fouquieria splendens* Engelm., *Neltuma glandulosa* (Torr.) Britton & Rose., *Vachellia constricta* (Benth.) Seigler & Ebinger, *V. farnesiana* (L.) Wight & Arn., and *Aloysia gratissima* (Gillies & Hook.) Tronkc, and the abundance of annual grasses, such as *Aristida adsencionis* L. and *Bouteloua barbata* Lag, as well as the introduced African species *Melinis repens* (Willd.) Zizka and *Cenchrus ciliaris* L.

### 3.2. Climatic Database and Edaphoclimatic Variables

With machine learning, a wide range of parameters can now be easily used in ENM studies, resulting, at times, in the use of duplicated or irrelevant variables in modeling. The careful selection of essential and relevant variables has thus been recommended [79,80]. Studies have shown that good predictive ability is possible even with only three variables [19,81].

In this study, we initially used 11 direct variables and 4 derived variables (Table 5). These variables were generated from the Instituto Nacional de Estadistica Geografia e Informatica (INEGI) climatic database with a vector climate dataset (minimum and average temperature and precipitation in monthly and annual averages) and soil-type data with a scale of 1:1,000,000 [82]. The altitude thematic layer of INEGI [83] was likewise used at a resolution of 1:10,000. The direct variables refer to those obtained directly from the digital edaphoclimatic layers (e.g., altitude, annual and monthly precipitation). The derived variables in this study were day and night mean annual and monthly temperatures, which were generated from the digital edaphoclimatic information in the following manner [84]:

Monthly average daytime temperature:

Td = Tm + [(Txm − Tim)(11 − To)]/4(12 − To)Sin[π((11 − To)/(11 + To))](1)

Monthly average night temperature:

Tn = Tm − [(Txm − Tim)(11 − To)]/4(12 − To)Sin[π((11 − To)/(11 + To))](2)

where

Td = Monthly average daytime temperature (°C)
Tn = Monthly average night temperature (°C)
Txm = Monthly average maximum temperature (°C)
Tim = Monthly average minimum temperature (°C)
Tm = Monthly average temperature (°C)
To = 12–0.5 N
N = Photoperiod (the value corresponding to the 15th of each month was used)
Sin = Sine expressed in radians
Π = 3.1416

The annual average daytime and nighttime temperatures were obtained by averaging the 12 monthly values of daytime and nighttime temperatures [84].

### 3.3. Model Construction and Calibration

A geographic information system (GIS) platform was used in the construction of the model; GIS tools make it possible to combine different types of data sources, carry out complex analyses, and generate maps [85,86]. The construction and calibration of the model involved a series of processes (Figure 1), the first of which was the characterization of the ecotype habitat in order to obtain a general description of the soil and climate factors influencing its distribution [87,88,89,90]. A projection of the ecotype collection site was made on each of the thematic layers, and each value was obtained using the Extract Multi Values to Points Tool of ArcGIS 10.3 software of Esri, Inc.^®^, Redlands, CA, USA. This resulted in a general data matrix integrated with the annual average and the maximum and minimum monthly values of the direct variables and the derived variables (Table 5). The maximum and minimum monthly values of each climatic variable were obtained from this general matrix. In order to establish ranges for the variables for the reclassification of the images and to overcome the limitation of having only a single record of the ecotype, we considered the mean true error of the digital layers of temperature and precipitation. According to their metadata, they had been extracted from WorldClim, so they were assumed to present the mean true error generated in the interpolation process, which is <10 mm or <10% for precipitation and <0.5 °C for temperature [91]. In determining the ranges of the variables, we thus considered a deviation of 10 mm for precipitation, while for temperature, the value was arbitrarily set to half of the usual value (0.25 °C), considering that the ecotype collection site was located on a plain with slopes of <2%. For soil and altitude, only the specific data of the collection site was obtained.

The Reclassify Tool in the Spatial Analyst Tools of ArcGIS was then used to generate images with data falling within a range defined for each variable. Each reclassified image was analyzed, observing the specificity or generality of each variable reflected on the images. The images that showed specificity were selected as key variables. From the overlay of these images, the two initial models were built using the Map Algebra in the Spatial Analysis Tools. In this way, Model A was built considering key direct variables, while Model B was built with key derived variables. Both models were converted into the KLM format for projection onto the Google Earth platform, which may be accessed through mobile devices. This enabled immediate access to the predicted population distribution area and its georeferenced location during the field validation stage.

### 3.4. Model Validation

The validation process involved comparing the model with a series of independent observations in the field in order to verify if it was developed as expected [92,93]. Since the study focused on modeling the potential population distribution of an understudied ecotype and not a species, it was necessary to develop a field work methodology that would make it possible to define in detail and systematize the validation process in the field. The fieldwork methodology that was developed and used in this study is as follows:

*Digital mesh generation for directed sampling*. A georeferenced digital mesh was generated that would allow, through the use of latitude–longitude data, specific strategic sites to be located and sampled in the areas projected by each of the two distribution models. The quadrant meshes were generated in a GIS environment. Prior to this process, the total surface area and geometric shape of each distribution model were first determined. Then, each mesh was constructed with a spacing of 100 ha between centroids. Hence, each quadrant, a possible sampling object, had its georeferenced data (latitude and longitude). The generated mesh used in field validation was also converted to the KLM format using the tool “layer to KLM in ArcGis Arctool Box” and projected on the Google Earth platform for access through mobile devices. Thus, with the digital layer, it was possible to visualize in the field not only the population distribution, as predicted by the model, but also the series of quadrants that covered the total predicted distribution area of the ecotype. This gave greater precision to the field validation of the presence (true positive) and absence (true negative) of the ecotype in each quadrant being sampled in the field.

*Field Sampling Strategy*. In the field, the Modified-Whittaker sampling method [94] was applied. The main advantage of this method is that it provides data that are less affected by spatial autocorrelation. The sampling method was systematically applied as follows:

(A) In the field, at least three georeferenced quadrants of 100 ha inside and outside of the predictive population distribution model were randomly selected from the digital mesh through the use of the Google Earth platform (which could be accessed in the field using a mobile device). A nested sampling strategy [94] was applied in each quadrant. This consisted of establishing, on each vertex of the quadrant and on the sides, a sampling sub-site. In this method, each quadrant had a total of eight sampling sub-sites with a longitudinal distance between sub-sites of approximately 3 ha. At each sub-site, sampling was performed following a circular route to cover an area of approximately 250 m in diameter, wherein an attempt was made to locate the ecotype that is the object of validation. Once located, the *B. curtipendula* specimens were verified for correct ecotype identification using ecotype-specific varietal descriptors established by the Sistema Nacional de Inspección y Certificación de Semillas [95].
(B) The data collected in each quadrant were geographical location (latitude and longitude), quadrant number, and model being sampled. For the sub-sites, the data format included geographical location and a general description of the conditions that each sub-site presented, including aspects such as the type of dominant vegetation, dominant species, slope, geographic exposure, and presence of livestock.
(C) Sampling was systematically applied to each distribution model, both in areas where the distribution of the ecotype was predicted and outside of the projected distribution areas. Overlapping areas in the two models were considered as predicted presence areas in both models. In areas where the non-presence of the ecotype was projected (i.e., outside of the predicted distribution area), sampling was similar to that in sub-sites of presence. The only difference was that the quadrants to be sampled were selected at a minimum distance of 450 m between the selected non-presence quadrant and where the model predicted the presence of the ecotype, i.e., they were sufficiently far from the distribution area predicted by the model.

The total number of quadrants and sub-sites subject to validation varied, based on the predicted potential surface area of the ecotype, as well as some logistical aspects that made sampling difficult, e.g., accessibility to the area, problems of insecurity in the site.

*Field Data Quality Analysis*. After the collection of field information, a quality analysis of the sampling sites was conducted. This involved projecting on the Google Earth platform both the model and the geographical location of each validation site in order to determine if there was a correspondence between the field sample sites and the model, i.e., to verify if each of the sites that were sampled corresponded to the distribution models being validated.

On the other hand, the validation sites that were outside the predicted distribution model were also projected in the same way as the sites inside the predicted distribution model. However, an extra analysis was carried out on these sites, which involved measuring the distance between the model and the georeferenced point of the reported site. This was done to check if the site met the pre-determined distance requirement (i.e., >450 m) between the distribution predicted by the model and the field site being sampled.

### 3.5. Sensitivity Analysis

A sensitivity analysis was conducted to identify which variables were most influential in driving the model’s behavior and to observe the performance of the variables as individual predictors. This part of the study was considered relevant from an agronomic perspective, since, by identifying the variables with the greatest predictive power, they can be used to select or prioritize potential rehabilitation areas.

The analysis was carried out considering the six variables making up Model A (the base model). GIS was used to estimate the surface area of the population distribution predicted by Model A with all six variables. Then, six submodels of Model A were generated (Figure 3), each of which excluded one of the six variables. The surface area of the population area predicted by each submodel was calculated and compared with that of Model A to see how the exclusion of the variable affected the distribution area.

The quadrants sampled during the field validation phase of Model A were projected onto each submodel, thereby making it possible to use the predicted presence/absence information (Table 2) to perform two statistical tests on each submodel: a negative LR test and True Skill Statistic (TSS).

To further observe the performance of the variables as individual predictors, they were projected individually (Figure 4) onto the Chihuahuan Desert area, where a collection site in Zacatecas was located. We checked if the Zacatecas collection site was within the distribution area projected by each variable.

### 3.6. Statistical Analysis

The non-parametric statistical tests applied in the present study were derived from the confusion matrix [92,96], also known as contingency matrix or error matrix [34]. This confusion matrix describes the frequency at which presence and absence sites are correctly and incorrectly predicted by the model. Thus, to evaluate the level of precision of the models, it was necessary to generate a confusion matrix identifying the true positives (a), false positives (b), false negatives (c), and true negatives (d) predicted by the model. From this confusion matrix, the following precision measures were calculated:

Overall Accuracy: This test quantifies the correct classification made by the model.

Overall Accuracy = (a + d)/n(3)

Error = 1 − Overall Accuracy(4)

Sensitivity: This test expresses the conditional probability that case X is correctly classified as true.

Sensitivity = a/a + c(5)

Specificity: The term “specificity” refers to the conditional probability that a case other than X will be correctly classified as false

Specificity = d/b + d(6)

Positive Likelihood Rate = Sensitivity/(1 − Specificity)(7)

Negative Likelihood Rate = (1 − Sensitivity)/Specificity(8)

True Skill Statistic = Sensitivity + Specificity − 1(9)

Positive Predictive Power: This test evaluates and quantifies the probability that a case is X if the algorithm classifies the case as X

Positive Predictive Power = a/a + b(10)

Negative Predictive Power: This determines the probability that a case is not X if the algorithm does not classify the case as X.

Negative Predictive Power = d/d + c(11)

Odds Ratio: This test quantifies the predictive power of the model beyond probability expectations [96,97]. It shows the relationship between correctly assigned cases and incorrectly assigned cases [98].

Odds Ratio = ad/cb(12)

where

a = correctly predicted presence data
b = incorrectly predicted presence data
c = incorrectly predicted absence data
d = correctly predicted absence data
n = sum of a + b + c + d

## 4. Conclusions

This research generated and validated an approach that can be used in the ENM of an ecotype, particularly when the greatest limitation is the small number of georeferenced sites available for modeling. This is the first study to describe the habitat of the B-31 ecotype of *B. curtipendula* in terms of the following variables: soil type, average minimum and maximum temperature in January; average maximum temperature in June; average minimum temperature in July; and average precipitation in August. Defining the climatic variables influencing an ecotype’s distribution can facilitate future studies on the impact of climate change on its populations. The B-31 model that was constructed and validated for use in grasslands in the state of Chihuahua predicted a distribution area of 18,158 ha in the state.

The sensitivity analysis results suggest that the variable soil type may be a dominant factor in limiting the ecotype’s distribution; also important is the climatic variable average minimum temperature in July. Considering soil type as a major predictor variable, the potential area for grassland rehabilitation in the Chihuahuan Desert using this genetic material was estimated to be 7,181,735 ha. However, further study with additional statistical analysis and field data from sites across the Chihuahuan Desert region is needed in this regard.

Considering the rapid degradation of important ecosystems and the loss of rare and understudied species, continuing efforts have to be made to advance knowledge, even in less-than-ideal settings. Despite its limitations, the GIS-based approach developed in this study yielded results that might help facilitate the well-targeted collection and use of the understudied B-31 ecotype in the state of Chihuahua. Further study is needed to explore the applicability of the approach to rehabilitation efforts in the Chihuahuan Desert region and to the study of rare and understudied ecotypes or species in other settings.

## Figures and Tables

**Table 1:** Variables that best describe the habitat of *Bouteloua curtipendula* (Michx.) Torr. ecotype B-31 in the grasslands of Chihuahua State, Mexico.

Model A (Model with Direct Variables)	Model B (Model with Derived Variables)
Max. Temp. in June = 31.8–32.3 °C ^¥^	Day Temp. in June = 27.3–27.8 °C ^¥^
Max. Temp. in January = 18.4–18.9 °C	Day Temp. in January = 14.8–15.3 °C
Min. Temp. in January = 0.9–1.4 °C	Night Temp. in January = 4.6–4.9 °C
Min. Temp. in July = 14.8–15.3 °C	Night Temp. in July = 19.2–19.7 °C
Ppt in August = 118–138 mm ^¥^	
Soil Type = Foezem	

^¥^ Temperature and precipitation data are monthly averages.

**Table 2:** Confusion matrix for the predicted population distribution model of *Bouteloua curtipendula* (Michx.) Torr. ecotype B-31 considering direct variables (Model A) and derived variables (Model B).

Model	Georeferenced Sites	
	Presence	Absence
**A**		
Presence	23	2
Absence	8	13
**B**		
Presence	25	5
Absence	8	10

**Table 3:** Results of statistical tests of precision and certainty of the predicted population distribution models of *Bouteloua curtipendula* (Michx.) Torr. ecotype B-31 in the grasslands of Chihuahua State, Mexico, considering direct (Model A) and derived variables (Model B).

Precision and Certainty Statistics	Model	
	A	B
Overall Accuracy	0.78	0.73
Error	0.22	0.27
Sensitivity	0.74	0.76
Specificity	0.87	0.67
Positive Likelihood Rate	5.56	2.27
Negative Likelihood Rate	0.30	0.36
Positive Predictive Power	0.92	0.83
Negative Predictive Power	0.62	0.56
Odds Ratio	19	6

**Table 4:** Predicted distribution area of the habitat of *Bouteloua curtipendula* (Michx.) Torr. ecotype B-31 in the arid and semiarid grasslands areas in Chihuahua State, Mexico, considering different environmental variables.

	Variables								
	Soil Type	T Max June	T Max January	T Min January	T Min July	Ppt August			
Model /Submodel	Foezem	31.8–32.3 °C	18.4–18.9 °C	0.9–1.4 °C	14.8–15.3 °C	118–138 mm	Negative LR ^¥^	TSS ^£^	Area (ha)
A							0.30	0.61	18,158
1							0.42	0.48	57,964
2							0.30	0.61	19,860
3							0.30	0.61	18,158
4							0.30	0.61	18,158
5							0.30	0.61	24,686
6							0.30	0.61	18,210

Note: Cells filled in gray indicate that the corresponding variables are included in the model or submodel. Model A contained all the variables that best described the ecotype habitat (Table 1). Therefore, it was considered the base model for the purpose of this analysis. ^¥^ Negative Likelihood Rate. ^€^ True Skill Statistic.

**Table 5:** Initial variables considered in the generation of population distribution models of *Bouteloua curtipendula* (Michx.) Torr. ecotype B-31 in the grasslands of Chihuahua State, Mexico.

Direct Variables	Derived Variables
Altitude (amsl)	Average Annual Day Temperature (°C)
Annual Maximum Temperature (°C)	Average Monthly Day Temperature (°C)
Monthly Maximum Temperature (°C)	Average Annual Night Temperature (°C)
Annual Minimum Temperature (°C)	Average Monthly Night Temperature (°C)
Monthly Minimum Temperature (°C)	
Annual Mean Temperature (°C)	
Monthly Mean Temperature (°C)	
Annual Precipitation (mm)	
Monthly Precipitation (mm)	
Soil Type	
Evapotranspiration (mm)	

## Data Availability

The raw data supporting the conclusions of this article will be made available by the authors upon request.
